# Analytical Model of the Connection Handoff in 5G Mobile Networks with Call Admission Control Mechanisms

**DOI:** 10.3390/s24020697

**Published:** 2024-01-22

**Authors:** Mariusz Głąbowski, Maciej Sobieraj, Maciej Stasiak

**Affiliations:** Faculty of Computing and Telecommunications, Poznan University of Technology, 60-965 Poznań, Poland; mariusz.glabowski@put.poznan.pl (M.G.); maciej.stasiak@put.poznan.pl (M.S.)

**Keywords:** 5G networks, analytical modeling, blocking probability, handoff, reservation mechanism, threshold mechanism

## Abstract

Handoff mechanisms are very important in fifth-generation (5G) mobile networks because of the cellular architecture employed to maximize spectrum utilization. Together with call admission control (CAC) mechanisms, they enable better optimization of bandwidth use. The primary objective of the research presented in this article is to analyze traffic levels, aiming to optimize traffic management and handling. This article considers the two most popular CAC mechanisms: the resource reservation mechanism and the threshold mechanism. It presents an analytical approach to occupancy distribution and blocking probability calculation in 5G mobile networks, incorporating connection handoff and CAC mechanisms for managing multiple traffic streams generated by multi-service sources. Due to the fact that the developed analytical model is an approximate model, its accuracy was also examined. For this purpose, the results of analytical calculations of the blocking probability in a group of 5G cells are compared with the simulation data. This paper is an extended version of our paper published in 17th ConTEL 2023.

## 1. Introduction

The 5G mobile network is being deployed in many countries around the world and is becoming an integral mobile communication technology. However, the ubiquity of 5G services requires the deployment of a large number of base stations, resulting in a significant number of handoff operations compared to previous generations of cellular networks [1,2,3]. Also, due to the use of the 5G network in Internet of Things (IoT) applications, where the 5G network will provide simultaneous wireless connections for hundreds of thousands of sensors, it will be necessary to find appropriate resources not only in a given cell but also in neighboring ones [4]. Low latency and expanded networks mean that 5G can reach 10 times more devices per square kilometer than 4G. Therefore, correct dimensioning of the system and optimization of the use of its resources are important [5,6]. For this reason, 5G networks, especially their mathematical modeling, are the focus of extensive scientific research [7,8,9,10,11,12]. Reference [7] introduces a versatile mathematical methodology to assess performance reliability improvement algorithms for 5G systems. The proposed approach considers the unique characteristics of the radio interface and the service process for sessions at mmWave/THz base stations (BSs). It can assess the performance of systems operating with multi-connectivity, resource reservation mechanisms, and priorities for various types of traffic with different service requirements. In [8], mathematical models for the information interaction process among a group of entities (IoT devices) and a base station within the 5G-IoT ecosystem were presented. In this study, the examined process is depicted as a queueing system, involving a stream of incoming requests specifying desired volumes of system resources and a stream of service signals. The reception of these service signals triggers a reevaluation of the initially allocated volumes of system resources for incoming requests. Reference [9] proposed new mathematical models that fit the emerging scenarios of wireless network deployment and maintenance. The authors also provided the design and implementation of a verification methodology for these models through the simulations provided. In [10], the authors introduced a multi-class service system with service rates obtained as a solution to the optimization problem, a Markovian arrival process, and state-dependent preemptive priorities. Reference [11] proposed a new optimization algorithm that corrects the coverage results and restores the true value of 5G coverage. Reference [12] outlined the planning for 5G coverage, initially using a conventional three-sector cell, and suggested an enhanced cell structure featuring six sectors. This updated configuration incorporates an advanced antenna system to deliver improved 5G coverage. Therefore, analytical modeling is used in many areas related to 5G networks.

One of many elements that can help to better optimize resource usage may be the handoff mechanism. Various variations of the handoff mechanism have been the subject of many studies in relation to 5G networks [3,13,14,15,16,17]. In [3], the authors focused on the issue of the distributed-handoff delay and the handover failure of mobile nodes in SDN-based networks. It proposes a hybrid clustering technique that reduces the scanning phase of the mobile node by minimizing the number of nodes that need to be scanned for handover. Reference [14] proposed a hybrid handover technique based on long-short term memory (LSTM) and support vector machine (SVM) for predictive handover. This mechanism significantly reduces handover latency in predictive handover while maintaining high prediction accuracy. In [15], a handover decision-making algorithm was proposed that integrates the dwelling-time prediction technique and the technique for order preference by similarity to ideal solution (TOPSIS). The algorithm presented reduces the number of unnecessary handovers in 5G heterogeneous networks. Reference [16] studies the downlink coverage and intercellular handoff for a two-tier 5G heterogeneous network (5G HetNet) under cost deployment. The path loss model was suggested in both cases, namely, line of site (LOS) and non-line of site (NLOS) with different path loss exponents. In [17], the authors presented the novel wolf-based power-optimized handoff (WbPOH) strategy for managing signal drops and power usage. The fitness process of the grey wolf facilitates continuous monitoring for forecasting the nearest incoming handoff signal and identifying work-free nodes.

Another element that improves the optimal use of resources is the CAC mechanism [18,19,20,21,22]. Designing CAC algorithms for 5G mobile networks is especially challenging given the limited and highly variable resources and the mobility of users encountered in such networks. In [18], the authors proposed a new CAC algorithm with an efficient handoff for 4G and 5G networks. Reference [19] presented a novel CAC scheme for VoIP in the context of network nodes using unmanned aerial vehicles (UAVs) as relays to a backhaul 5G network. This scheme intercepts VoIP call control messages and decides on the admission of every new call based on a prediction of the network congestion level. A new CAC function, presented in [20], was also developed to adjust thresholds during handoff request signaling. To perform the handoff operation, the Markov chain technique was used to analyze the call blocking probability characteristic and decide handoff approval for various subscriber requests. In [21], the authors formulated a problem into a time series prediction task and employed a data-driven approach using two state-of-the-art machine learning concepts, namely deep neural networks (DNNs) and long short-term memory (LSTM) recurrent networks, to minimize the amounts of over-/under-reservations within a short-time prediction context.

Dimensioning mobile systems must consider all techniques and mechanisms that affect traffic control and the distribution of traffic offered to these systems. In the case of cellular wireless systems, the most significant mechanisms affecting the dimensioning of wireless systems (cells, sectors) are handover and CAC mechanisms. Dimensioning techniques for telecom systems use both analytical and simulation approaches. Analytical techniques require the development of occupancy probability distributions in these systems and blocking probabilities. This article aims to analyze and model the influence of connection handoff on the blocking probabilities of specific traffic classes (services) in the 5G mobile networks with CAC mechanisms. It is assumed that the offered traffic is generated by multi-service sources [13,23], i.e., it is assumed that each source can generate streams of different traffic classes. In the article, the authors focused on the two most popular CAC mechanisms: the resource reservation mechanism and the threshold mechanism [24,25,26,27].

Thus far, analytical models of 3G systems have been considered and presented in the literature [25,28]. They made it possible to determine the traffic characteristics of the 3G network systems considered. Additionally, analytical models that can be used in 4G network modeling and optimization processes were considered in the literature [29,30]. On the other hand, within the 5G network, cells are smaller in size, and the problem of handoff connections with high mobility of end users becomes very important.

The model developed in this paper takes into account the possibility that, in 5G networks, a specific call (a traffic stream of a given class) may be handled by one of the neighboring cells, not necessarily by the cell in which the call originated. The model is not dedicated to a specific 5G network implementation technology. It allows its use in the dimensioning process (determining the size of the necessary resources, e.g., bandwidth) of the system. As the developed model does not take into account the limitations and capabilities of specific devices/technologies, it cannot be used in further stages of the 5G system design, i.e., stages where specific technical solutions are already selected. However, the results obtained from the proposed model provide a basic source of information about the numerical dependencies between the volume of traffic offered by different classes of traffic, the desired quality parameters, and the amount of necessary resources in a system where the handling of a given request is allowed to be transferred to a neighboring resource, i.e., 5G systems.

The contributions of this article are summarized below:

- Firstly, this article presents a generalized model of the limited-availability group that can be used to determine the blocking probability for individual classes of requests offered in 5G systems without CAC mechanisms introduced.
- Secondly, this article describes a model of a limited-availability group with resource reservation mechanisms for blocking probability calculations in 5G systems with reservation mechanisms.
- Then, this article proposes a model of a limited-availability group with threshold mechanisms used for blocking probability calculations in 5G systems.
- Finally, this article presents the algorithm for a blocking probability calculation in the group of cells in 5G systems with CAC mechanisms.

The remainder of this article is structured as follows. Section 2 describes the basics of the analytical model; that is, the structure of the offered traffic and the limited-availability group model used to model a set of cells. In Section 4, the model of the group of 5G cells is presented. The numerical examples and the analysis of results are described in Section 5. This article ends with Section 6, which presents the most important conclusions resulting from the study carried out.

## 2. Limited-Availability Group

### 2.1. Generalized Model of the Limited-Availability Group with Erlang Traffic Streams

**Figure 1:**
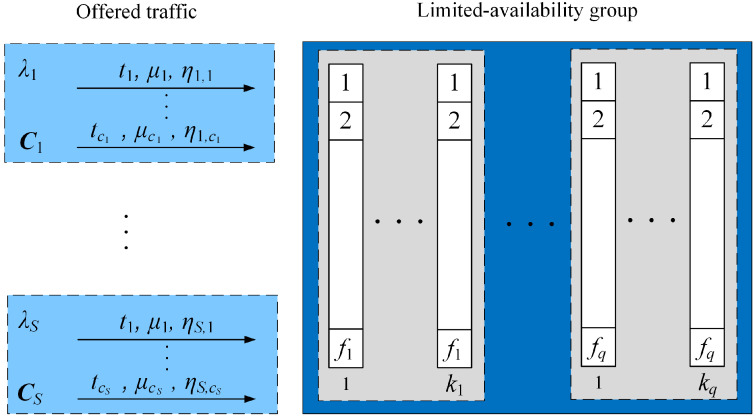
Generalized model of the limited-availability group.

Let us consider the generalized model of a limited-availability group (LAG); that is, the group consisting of separate transmission links (subgroups) with different capacities. Furthermore, assume that the system is made up of links of *q* types. Each type of link is explicitly identified by the following parameters: $k_{q}$—the number of links of type *q*; $f_{q}$—the capacity of links of type *q* (Figure 1). So, the total capacity *V* for the group is

(1) $$V=\sum_{s=1}^{q}k_{s}f_{s}.$$

The considered system services a call only if it can be completely handled by the resources of a single link.

### 2.2. Structure of Offered Traffic

In the model, there are defined *m* traffic classes. A call of class *c* belonging to the set M={1,2,…,m} requires $t_{c}$ basic bandwidth units (BBUs) (by a single BBU, we mean the greatest common divisor of requested resources (e.g., bandwidth) by calls of a given class of service) to establish the connection. The exponential service rate for class *c* calls is $\mu_{c}$.

The group presented in Figure 1 offers Erlang traffic streams. The given traffic stream *s* is generated by sources that belong to the appropriate set of traffic sources $Z_{s}$. The system defines *S* sets of traffic sources that generate Erlang traffic streams. The sources belonging to the set $Z_{s}$ can generate calls from the set $C_{s}=\left\{1,2,\ldots ,c_{s}\right\}$ according to the available set of services.

The share of class *c* (from set M) in the structure of traffic generated by the sources from the $Z_{s}$ set is determined by the $\eta_{s,c}$) parameter. For individual sets of Erlang traffic sources, this parameter satisfies the following relations:

(2) $$\sum_{c=1}^{c_{s}}\eta_{s,c}=1.$$

To determine the value of traffic $A_{s,c}$ offered by Erlang sources belonging to the set $Z_{s}$, we use the following formula:

(3) $$A_{s,c}=\eta_{s,c}\lambda_{s}/\mu_{c},$$

where $\lambda_{s}$ is the intensity of new calls generated by multi-service sources belonging to the set *s*.

### 2.3. Blocking Probability Calculations

An approximate method for calculating the blocking probability was proposed for the generalized model of the limited-availability group in [31]. According to this method, the occupancy distribution in the system is determined by the generalized Kaufman–Roberts recursion [32,33]:

(4) $$n{\left[P_{n}\right]}_{V}=\sum_{s=1}^{S}\sum_{c=1}^{m}A_{s,c}t_{c}\sigma_{c}\left(n-t_{c}\right){\left[P_{n-t_{c}}\right]}_{V},$$

where ${\left[P_{n-t_{c}}\right]}_{V}=0$, if $n<t_{c}$, and the value ${\left[P_{0}\right]}_{V}$ results from the normalizing condition:

(5) $$\sum_{n=0}^{V}{\left[P_{n}\right]}_{V}=1.$$

In Formula (4), the parameter ${\left[P_{n}\right]}_{V}$ is the state probability, i.e., the probability of the event that there are *n*-occupied BBUs in the system, and $\sigma_{c}\left(n\right)$ is the probability of admission of the class *c* call to the service when the system is found in the state *n*. Equation (4) results directly from the first work concerning the so-called full-availability group (a system with a complete sharing policy) with multi-rate traffic, i.e., [34,35,36,37]. If $\sigma_{c}\left(n\right)=1$, for each state of a given system, then the generalized Kaufman–Roberts recursion (4) is reduced to the Kaufman–Roberts recursion [35,36].

The conditional probability of passing $\sigma_{c}\left(n\right)$ for the class *c* traffic stream in the generalized model of the limited-availability group, characterized by the parameters *q*, $k_{q}$, $f_{q}$, and *V*, is determined with the assumption that—in the considered group—*n* BBUs are busy. According to the considerations presented in [31], the conditional probability of passing $\sigma_{c}\left(n\right)$ for the class *c* stream in the generalized model of the limited-availability group with parameters *q*, $k_{q}$, $f_{q}$, and *V*, can be determined based on the following formula:

(6) $$\sigma_{c}\left(n\right)=1-\frac{F(V-n,k_{1}\ldots k_{q},t_{c}-1)}{F(V-n,k_{1}\ldots k_{q},f_{1}\ldots f_{q})}.$$

Parameter $F(V-n,k_{1}\ldots k_{q},f_{1}\ldots f_{q})$ in Formula (6) expresses the number of possible arrangements of V−n-free BBUs in all subgroups, making up the limited-availability group, and $F(V-n,k_{1}\ldots k_{q},t_{c}-1)$—the number of such arrangements of BBUs in which the class *c* call cannot be serviced by any of the subgroups. So, the parameter $\sigma_{c}\left(n\right)$, determined according to Formula (6), is the probability of such arrangements of free BBUs in the state occupancy *n* in which the class *c* call can be serviced.

The value of the combinatorial function $F(x,k_{1}\ldots k_{q},f_{1}\ldots f_{q})$ in Formula (6), determining the number of possible arrangements of *x*-free BBUs in the limited-availability group, composed of links of *q* types, is determined—according to [31]—by the following formula:

(7) $$\begin{array}{c} F(x,k_{1},k_{2},\ldots ,k_{q},f_{1},f_{2},\ldots ,f_{q})=\\ \;\;\;\;\;=\sum_{x_{1}=0}^{x}\sum_{x_{2}=0}^{x-x_{1}}\cdots \sum_{x_{q-1}=0}^{x-\sum_{r=1}^{q-2}x_{r}}F\left(x_{1},k_{1},f_{1}\right)\cdot F\left(x_{2},k_{2},f_{2}\right)\cdot \cdots \cdot \\ \;\;\;\;\;\;\;\;\;\;\;\;\;\;\cdot F\left(x_{q-1},k_{q-1},f_{q-1}\right)\cdot F\left(x-\sum_{r=1}^{q-1}x_{r},k_{q},f_{q}\right), \end{array}$$

where F(x,k,f) defines the number of possible arrangements of *x*-free BBUs in *k* links (subgroups), and each one has a capacity equal to *f* BBUs, i.e., in the limited-availability group composed of links of one type [31]:

(8) F(x,k,f)=∑i=0⌊xf+1⌋(−1)ikix+k−1−i(f+1)k−1.

After determining $\sigma_{c}\left(n\right)$ (Formula (6)), we can calculate the occupancy distribution ${\left[P_{n}\right]}_{V}$ according to (4) and, subsequently, the blocking probability for calls from class *c*. The blocking state occurs when no link has a sufficient number of free BBUs to service class *c* calls. This means that for links of type *q*, any state in which the number of busy BBUs in each link is higher than $n=f_{q}-t_{c}$ (i.e., $f_{q}-t_{c}+1\leq n\leq f_{q}$) is the blocking one. All the possible blocking states in the limited-availability group composed of links of *q* types are determined by the following conditions:

(9) $$V-\sum_{s=1}^{q}k_{s}\left(t_{c}-1\right)\leq n\leq V.$$

Based on the calculated values of the conditional probabilities of passing $\sigma_{c}\left(n\right)$ (Equation (6)) and the occupancy distribution ${\left[P_{n}\right]}_{V}$ (Equation (4)), the blocking probability for calls of class *c* can be determined with the following formula:

(10) $$E_{c}=\sum_{n=V-\sum_{s=1}^{q}k_{s}\left(t_{c}-1\right)}^{V}{\left[P_{n}\right]}_{V}\left[1-\sigma_{c}\left(n\right)\right].$$

To summarize, the algorithm of determination of the occupancy distribution in the limited-availability group with multi-service Erlang streams, generated by multi-service sources, can be written as follows:

1. Determination of values of offered traffic $A_{s,c}$ based on (3).
2. Calculation of the values of the conditional passing coefficients based on (6).
3. Determination of state probabilities ${\left[P_{n}\right]}_{V}$ using (4).
4. Determination of blocking probabilities $E_{c}$ for calls to particular traffic classes using (10).

## 3. Limited-Availability Group with CAC Mechanisms

### 3.1. Resource Reservation Mechanism

Let us consider a generalized model of a limited-availability group to which multi-service traffic is offered. This group consists of *q* subgroups. Each subgroup contains $k_{q}$ links with a capacity of $f_{q}$ each. In a limited-availability group with a total capacity of *V* BBUs (Equation (1)), let us introduce a $Q_{c}$ reservation limit for individual call classes. The value of the $Q_{c}$ parameter determines the limit state in which it is possible to start handling class *c* requests. The reservation threshold $Q_{c}$ is introduced only for traffic classes belonging to the $R=\{1,2,\ldots ,m_{R}\}$ set, which is a subset of the set of all traffic classes M={1,2,…,m}. The remaining classes are treated as privileged.

The $R_{c}$ reservation area includes all states above the border state $Q_{c}$. The size of the $R_{c}$ reservation area can be determined by the following formula:

(11) $$R_{c}=V-Q_{c}.$$

In the $R_{c}$ reservation area, class *c* calls will be blocked.

If the value of the $Q_{c}$ reservation limit meets the following conditions:

(12) $$V-t_{c}\leq Q_{c}\leq V,$$

then the value of the $Q_{c}$ parameter will have no impact on the service process.

However, if the reservation areas $R_{c}$ of individual classes, belonging to the set R, are equal and meet the following conditions:

(13) $$R_{c}>t_{max},$$

where $t_{max}$ is the maximum number of BBUs requested by the calls of the so-called “oldest” class (the class that requests the most BBUs to handle the call), then the blocking probabilities $E_{c}$ (c∈M) of calls of each class will also be equal [32].

**Figure 2:**
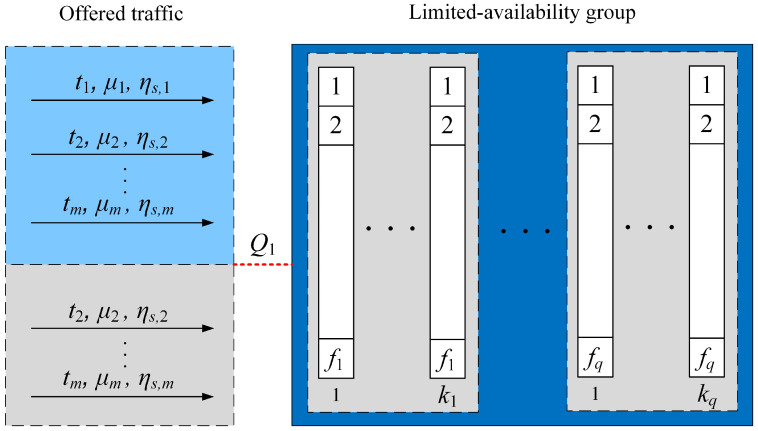
Model of the limited-availability group with reservation mechanisms. Class 1 belongs to the set R.

The system considered, presented in Figure 2, allows service calls of class *c* (belonging to set R) only if a call can be fully handled by the resources of a single link. Additionally, the system admits such calls when the number of BBUs available in the group is greater than or equal to the specified reservation area value, $R_{c}$. Calls of class *c* that do not belong to the set R are allowed for service only if they can be handled entirely by the resources of a single link. It is important to note that in systems with limited-availability, the resources required to service a single call cannot be spread over multiple links, distinguishing this characteristic feature [31]. Therefore, a limited-availability group serves as an illustrative example of a system exhibiting state-dependent service processes, where the state dependence arises from the group’s structure and the introduced reservation mechanism.

To reflect the influence of a specific structure of a group and an introduced reservation mechanism on the process of determining the occupancy distribution, we need to take into account two conditional transition coefficients, respectively. The first, $\sigma_{c}\left(n\right)$, is related to the specific structure of the group, expressed by (6). The second conditional transition coefficient, $\sigma_{c,R}\left(n\right)$, takes into account the impact of introducing the reservation mechanism on the service process and can be expressed in the following formula:

(14) $$\sigma_{c,R}\left(n\right)=\left\{\begin{array}{cc} 1 & \mathrm{for}\;n⩽R_{c}\wedge c\in R,\\ 0 & \mathrm{for}\;n>R_{c}\wedge c\in R,\\ 1 & \mathrm{for}\;c\notin R. \end{array}\right.$$

It should be noted that the reservation mechanism presented is implemented in the limited-availability group, regardless of its structure. This approach allows for the utilization of a comprehensive description of the total transition coefficient in the limited-availability group:

(15) $$\sigma_{c,\mathrm{Tot}}\left(n\right)=\sigma_{c}\left(n\right)\cdot \sigma_{c,R}\left(n\right).$$

The next step is to include both dependencies in (4) determining the occupancy distribution in the group with limited availability and the reservation mechanism. Therefore, (4) should be rewritten into the following form:

(16) $$n{\left[P_{n}\right]}_{V}=\sum_{s=1}^{S}\sum_{c=1}^{m}A_{s,c}t_{c}\sigma_{c,\mathrm{Tot}}\left(n-t_{c}\right){\left[P_{n-t_{c}}\right]}_{V}.$$

After calculating the occupancy distribution of ${\left[P_{n}\right]}_{V}$ in the limited-availability group with a reservation mechanism, the blocking probability for calls of class *c* belonging to the set mathbbM can be determined as follows:

(17) $$E_{c}=\sum_{n=V-\sum_{s=1}^{q}k_{s}\left(t_{c}-1\right)}^{V}{\left[P_{n}\right]}_{V}\left[1-\sigma_{c,\mathrm{Tot}}\left(n\right)\right].$$

In conclusion, the algorithm for determining the occupancy distribution in a limited-availability group with multi-service Erlang streams, generated by multi-service sources, and with the reservation mechanism, can be formulated as follows:

1. Determination of values of the offered traffic $A_{s,c}$ according to (3).
2. Calculation of the values of the total conditional passing coefficients based on (15).
3. Determination of state probabilities ${\left[P_{n}\right]}_{V}$ on the basis of the modified Kaufman–Roberts recursion (16).
4. Determination of the blocking probabilities $E_{c}$ for calls of particular traffic classes using (17).

### 3.2. Threshold Mechanism

**Figure 3:**
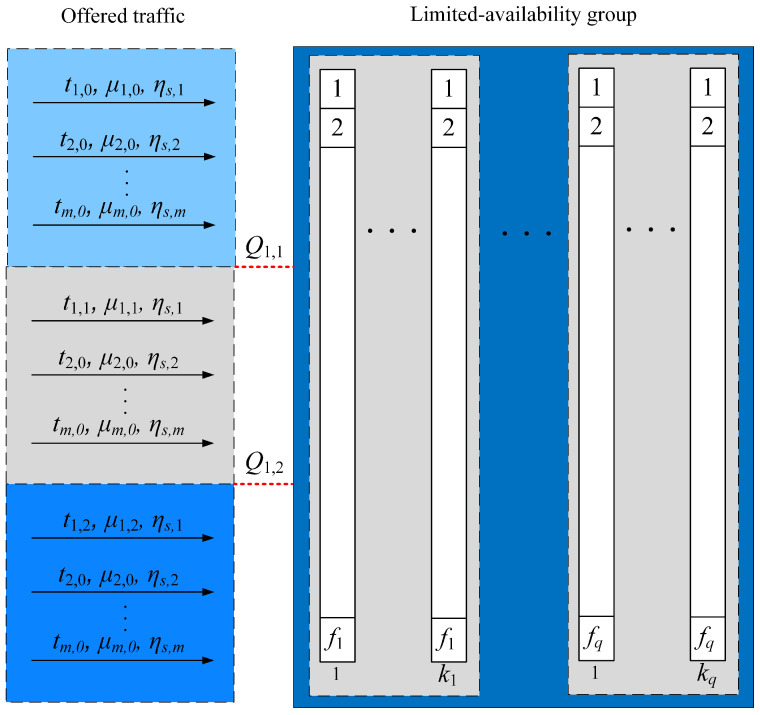
Model of the limited-availability group with the threshold mechanism. Class 1 belongs to the set T.

Let us consider another state-dependent system model, i.e., the limited-availability group with multi-service traffic, in which the offered traffic parameters may change depending on the occupancy of system resources. An example of such a system may be a multi-threshold system. In this model, we assume that for each class *c* call that belongs to the T set, a set of $p_{c}$ thresholds $\left\{Q_{c,1},Q_{c,2},\ldots ,Q_{c,p_{c}}\right\}$ is introduced individually, where the first index determines the call class and the second index determines the threshold number. Additionally, it is assumed that $\left\{Q_{c,1}\leq Q_{c,2}\leq \ldots \leq Q_{c,p}\right\}$. Figure 3 shows the class 1 traffic stream offered in the pre-threshold area $\left(0\leq n\leq Q_{1,1}\right)$, the post-threshold area 1 $\left(Q_{1,1}\leq n\leq Q_{1,2}\right)$, and the post-threshold area 2 $\left(Q_{1,2}\leq n\leq V\right)$, respectively. It should be noted that the threshold area *u* is the inter-threshold area bounded by thresholds $Q_{c,u}$ and $Q_{c,u+1}$.

The operation of a multi-threshold system can be presented as follows: In each post-threshold area *u* of class *c*, a traffic stream of class *c* is offered, which is determined by its own set of parameters $\left\{t_{c,u},\mu_{c,u}\right\}$, where $t_{c,u}$ represents the number of BBUs requested by the class *c* call in the post-threshold area *u*, and $\mu_{c,u}$ is the service stream intensity (the inverse of the average service time) of class *c* in the post-threshold area *u*. Additionally, it is assumed that $t_{c,0}>t_{c,1}>\ldots >t_{c,u}>\ldots >t_{c,p_{c}}$ and $\mu_{c,0}^{-1}<\mu_{c,1}^{-1}<\ldots <\mu_{c,u}^{-1}<\ldots <\mu_{c,p_{c}}^{-1}$. This means that as the load on the group increases, the number of requested BBUs required to handle calls of particular classes decreases and, at the same time, the service time may increase.

The considered limited-availability group with threshold mechanisms offers Erlang traffic streams. In order to include the influence of the threshold mechanisms on the traffic value $A_{s,c,u}$, generated by Erlang sources belonging to the set $Z_{s}$ in the threshold area *u*, Formula (3) is to be adequately modified:

(18) $$A_{s,c,u}=\eta_{s,c}\lambda_{s}/\mu_{c,u}.$$

Observe that in the model of the limited-availability group with multi-service traffic sources and threshold mechanisms, the operation of the threshold mechanisms introduces an additional dependence between the service stream in the system and the current occupancy state of the system. To include this dependence in the considerations, in (4), we introduce the coefficient $\sigma_{c,u,T}\left(n\right)$ that determines the occupancy states in the system in which the offered traffic is defined by the parameters $\left\{t_{c,u},\mu_{c,u}\right\}$. Notice that when examining the model involving a limited-availability group with multi-service traffic sources and threshold mechanisms, the presence of threshold mechanisms creates an extra connection between the service stream within the system and the current occupancy state. In order to incorporate this relationship into our analysis, we will incorporate a coefficient, denoted as $\sigma_{c,u,T}\left(n\right)$. This coefficient is crucial in determining the occupancy states of the system where the offered traffic is defined by parameters $t_{c,u},\mu_{c,u}$.

(19) $$\sigma_{c,u,T}\left(n\right)=\left\{\begin{array}{cc} 1 & \mathrm{for}\;Q_{c,u}<n\leq Q_{c,u+1},\\ 0 & \mathrm{for}\;\mathrm{remaining}\;n. \end{array}\right.$$

In addition, changing volumes of resources that are allocated to calls in particular occupancy states impose a change in the way the conditional transition coefficient $\sigma_{c}$ is determined (Formula (6)), i.e., the coefficient that describes the influence of the structure of the system on the new call admission process:

(20) $$\sigma_{c}\left(n\right)=1-\frac{F(V-n,k_{1}\ldots k_{q},t_{c,u}-1)}{F(V-n,k_{1}\ldots k_{q},f_{1}\ldots f_{q})},$$

where the value of function $F(V-n,k_{1}\ldots k_{q},t_{c,u}-1)$ is determined by Formula (7). The value $t_{c,u}$ is matched according to the number of thresholds *u* determined on the basis of state *n*.

Observe that the threshold mechanisms are introduced to the group regardless of its structure, which allows for a product form description of the total transition coefficient in the limited-availability group:

(21) $$\sigma_{c,u,\mathrm{Tot}}\left(n\right)=\sigma_{c}\left(n\right)\cdot \sigma_{c,u,T}\left(n\right),$$

Then, the occupancy distribution in the considered threshold system can be calculated as follows:

(22) $$n{\left[P_{n}\right]}_{V}=\sum_{s=1}^{S}\sum_{c=1}^{m}\sum_{u=0}^{p_{c}}A_{s,c,u}\sigma_{c,u,\mathrm{Tot}}\left(n-t_{c,u}\right)t_{c,u}{\left[P_{n-t_{c,u}}\right]}_{V}.$$

The determined occupancy distribution allows us to determine the blocking probability for each of the *m* service classes. For calls of class *c*, this can be expressed by the following formula:

(23) $$E_{c}=\sum_{n=V-t_{c,p_{c}}+1}^{V}{\left[P_{n}\right]}_{V}\left(1-\sigma_{c}\left(n\right)\right).$$

In summary, the process of establishing the occupancy distribution in the multi-threshold limited-availability group featuring multi-service Erlang streams, originating from multi-service sources, can be articulated in the following manner:

1. Determination of values of offered traffic $A_{s,c,u}$ for threshold area *u* according to (18).
2. Calculation of the values of the total conditional passing coefficients for threshold area *u* based on (21).
3. Determination of state probabilities ${\left[P_{n}\right]}_{V}$ on the basis of the modified Kaufman–Roberts recursion (22).
4. Determination of the blocking probabilities $E_{c}$ for particular traffic class calls using (23).

## 4. Traffic Flows Optimization in 5G Networks

**Figure 4:**
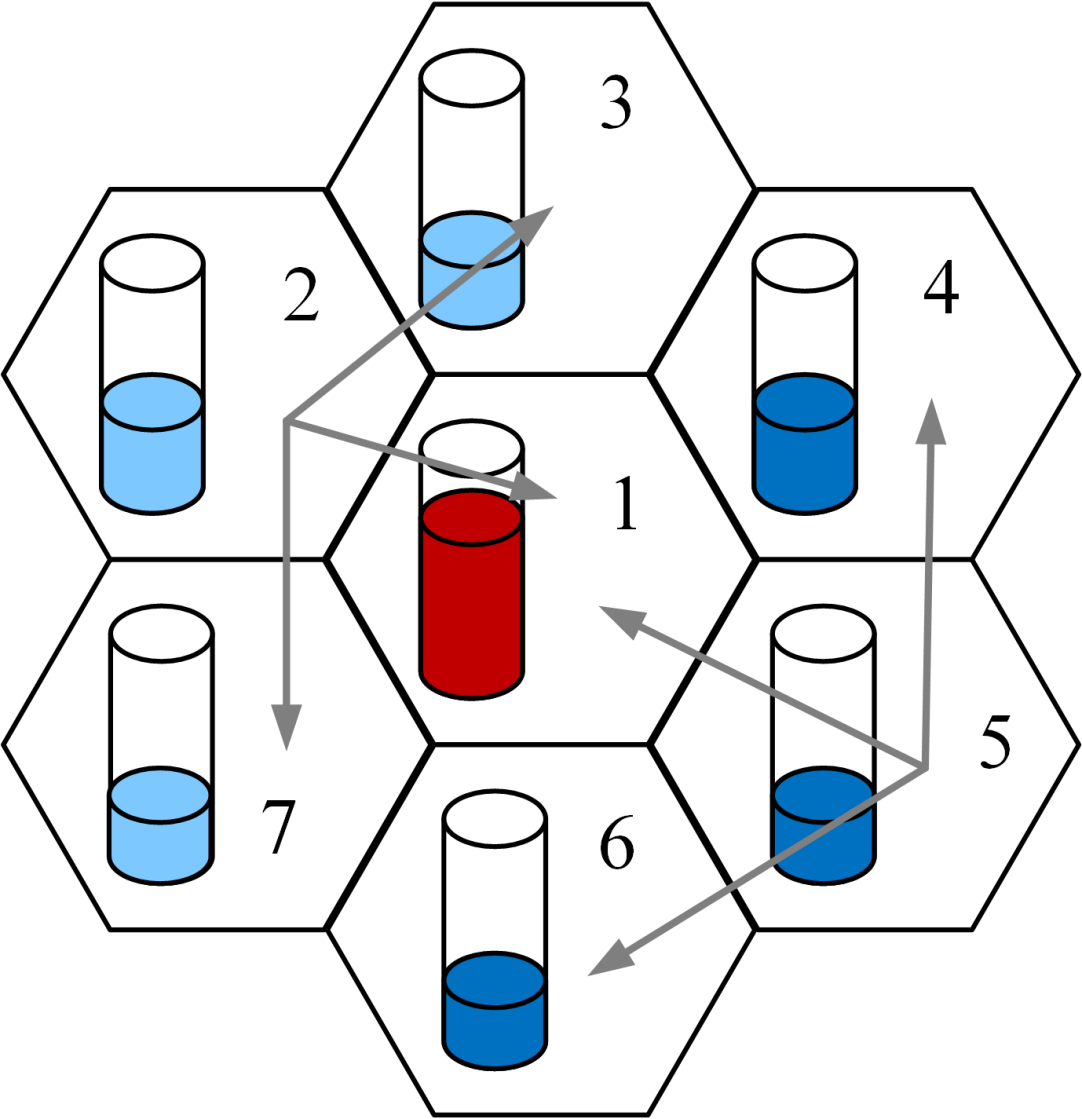
Connection handoff in a group of cells.

A cluster of cells equipped with a connection handover mechanism can be conceptualized as a system designed to optimize connection arrangements within a specific cell, maximizing the utilization of group resources [25,28]. Figure 4 illustrates a set of seven chosen cells, each featuring a symbolic vessel reflecting the radio interface load level. Cells 1, 2, and 4 exhibit higher loads. The handover mechanism facilitates the redirection of incoming calls, which cannot be accommodated in cells 1, 2, and 4, to neighboring cells—such as from cell 2 to cells 3 and 7. This system’s operation reflects that of a limited-availability group, where an individual cell corresponds to a subgroup within the group. If a specific subgroup (cell) cannot manage a connection, but there are ample resources in other subgroups of the group, the connection is directed to one of those suitable subgroups.

Then, consider a group of such cells, each with a different capacity. Assume that the term “group of cells” represents the set of all cells in a given area under consideration, and the term “assembly of cells” represents the set of cells that are directly (to each other) adjacent. Assume that a group of cells offers *S* Erlang traffic streams. A group of cells services a call only if one of the cells can fully service it. Notice that a call arriving in a defined cell will be admitted if either that cell or any other cell in its direct neighborhood (a defined assembly of cells) has a sufficient number of resources available.

Based on the above, define the traffic characteristics of the cell group considering the hard handoff mechanism (in a hard handoff mechanism, there is an actual break in the connectivity while switching from one cell to another). These characteristics can be derived using the generalized model of the limited-availability group, which will be used to model the considered cell system. In [25,28], a method for modeling a group of cells that jointly service (using a connection handoff mechanism) streams of integrated traffic was proposed. For every cell, we define adjacent cells, and such an assembly of cells will be modeled with the limited-availability group. For example, for a group of cells presented in Figure 4, we will perform calculations for K=7 assemblies of adjacent cells (*K*—the number of assemblies):

- cell 1 and its neighboring cells: 2, 3, 4, 5, 6, 7;
- cell 2 and its neighboring cells: 3, 1, 7;
- cell 3 and its neighboring cells: 4, 1, 2;
- cell 4 and its neighboring cells: 5, 1, 3;
- cell 5 and its neighboring cells: 6, 1, 4;
- cell 6 and its neighboring cells: 7, 1, 5;
- cell 7 and its neighboring cells: 2, 1, 6;

In the case of each cell assembly, we establish its configuration, which encompasses the capacities of individual cells, along with the corresponding offered traffic value contributing to the overall traffic load on a group of cells. Subsequently, for each assembly, we analyze both the occupancy distribution and the blocking probability. The resulting blocking probability values for each assembly serve as input data for calculating the overall blocking probability across the entire area.

In reference [28], two heuristic methods are proposed that allow the probability of blocking in the whole area (group of cells) to be determined based on the probabilities of blocking in particular assemblies of cells. According to the first method, this probability is defined as the geometric mean of the probabilities of particular assemblies of cells, and in the other method, it is defined as the weighted mean, with weights being the values of traffic offered to particular assemblies of cells.

Now, consider the assembly of cells with a capacity of $V_{g}$ BBUs that offer *S* Erlang traffic streams. The mean Erlang traffic offered by class *c* from set *s* to the *g* cell assembly depends on CAC mechanisms and can be determined as follows:

- In the case of a system without CAC mechanisms or with a reservation mechanism:
  (24) $$A_{s,c}^{g}=\frac{\eta_{s,c}\lambda_{s}^{g}}{\mu_{c}},$$
- For a system with the following threshold mechanism:
  (25) $$A_{s,c,u}^{g}=\frac{\eta_{s,c}\lambda_{s}^{g}}{\mu_{c,u}},$$
  where $\lambda_{s}^{g}$ is the mean intensity of the *s* call stream in the *g* cell assembly

For all call streams, we assume that the offered traffic is evenly distributed across all cells.

Having $A_{s,c}^{g}$ or $A_{s,c,u}^{g}$ traffic values, the generalized Kaufman–Roberts recursion for assembly *g* can be rewritten in the form that includes characteristics of Erlang traffic streams generated by multi-service traffic sources, namely:

- In the case of a system without CAC mechanisms:
  (26) $$n{\left[P_{n}\right]}_{V_{g}}=\sum_{s=1}^{S}\sum_{c=1}^{m}A_{s,c}^{g}\sigma_{c}^{g}\left(n-t_{i}\right)t_{i}{\left[P_{n-t_{i}}\right]}_{V_{g}},$$
- In the case of a system with reservation mechanisms:
  (27) $$n{\left[P_{n}\right]}_{V_{g}}=\sum_{s=1}^{S}\sum_{c=1}^{m}A_{s,c}^{g}\sigma_{c,\mathrm{Tot}}^{g}\left(n-t_{i}\right)t_{i}{\left[P_{n-t_{i}}\right]}_{V_{g}},$$
- In the case of a system with threshold mechanisms:
  (28) $$n{\left[P_{n}\right]}_{V_{g}}=\sum_{s=1}^{S}\sum_{c=1}^{m}\sum_{u=0}^{p_{c}}A_{s,c,u}^{g}\sigma_{c,u,\mathrm{Tot}}^{g}\left(n-t_{i}\right)t_{i}{\left[P_{n-t_{i}}\right]}_{V_{g}}.$$

Next, based on occupancy distribution ${\left[P_{n}\right]}_{V_{g}}$, we can calculate the blocking probability $E_{c}^{g}$ for class *c* calls in assembly *g*:

- In the case of a system without CAC mechanisms:
  (29) $$E_{c}^{g}=\sum_{n=V_{g}-\sum_{s=1}^{q}k_{s}\left(t_{c}-1\right)}^{V_{g}}{\left[P_{n}\right]}_{V_{g}}\left[1-\sigma_{c}^{q}\left(n\right)\right].$$
- In the case of a system with reservation mechanisms:
  (30) $$E_{c}^{g}=\sum_{n=V_{g}-\sum_{s=1}^{q}k_{s}\left(t_{c}-1\right)}^{V_{g}}{\left[P_{n}\right]}_{V_{g}}\left[1-\sigma_{c,\mathrm{Tot}}^{g}\left(n\right)\right].$$
- In the case of a system with threshold mechanisms:
  (31) $$E_{c}^{g}=\sum_{n=V_{g}-t_{c,p_{c}}+1}^{V_{g}}{\left[P_{n}\right]}_{V_{g}}\left(1-\sigma_{c}^{g}\left(n\right)\right).$$

The achieved values of blocking probability for each assembly make up input data for determining blocking probability in the whole area. Below, we present two methods that make it possible to determine the traffic characteristics of the system considered:

- Method 1:
  (32) $$E_{c}=\sqrt[{G}]{E_{c}^{1}\cdot E_{c}^{2}\cdot \cdot \cdot E_{c}^{G}},$$
  where *G* denotes the number of assemblies of cells in a given group of cells (in the considered area).
- method 2:
  (33) $$E_{c}=\sum_{g=1}^{G}E_{c}^{g}\cdot w_{c}^{g},$$
  where $w_{c}^{g}$ denotes part of the total traffic of class *c* offered to assembly *g*:
  (34) $$w_{c}^{g}=\frac{\sum_{s=1}^{S}A_{s,c}^{g}}{\sum_{s=1}^{S}\sum_{c=1}^{m}\sum_{g=1}^{G}A_{s,c}^{g}}.$$
  In the case of a system with a reservation mechanism, we apply (34) directly. However, in the case of a system with a threshold mechanism, we have to substitute $A_{s,c}^{g}$ with $A_{s,c,0}^{g}$ in (34).

The process of establishing the occupancy distribution and the blocking probability in the considered 5G system featuring multi-service Erlang streams, originating from multi-service sources, can be articulated in the following manner:

1. Setting the number of assemblies to g=1.
2. Determination of values of offered traffic $A_{s,c}^{g}$ for assembly *g* according to (24) or (25), depending on the introduced CAC mechanism.
3. Calculation of the values of the total conditional passing coefficients for assembly *g* based on (6), (15), or (21), depending on the introduced CAC mechanism.
4. Determination of state probabilities ${\left[P_{n}\right]}_{V}^{g}$ for assembly *g* on the basis of the modified Kaufman–Roberts recursion (26), (27), or (28), depending on the introduced CAC mechanism.
5. Determination of the blocking probabilities $E_{c}^{g}$ for particular traffic class calls in assembly *g* using (29), (30), or (31), depending on the introduced CAC mechanism.
6. Increasing the number of assemblies to g=g+1.
7. Checking the assembly number. If g≤7, go to Step 2.
8. Calculation of the blocking probabilities $E_{c}$ for particular traffic classes in the considered 5G system using (32) (Method 1) or (33) (Method 2).

## 5. Numerical Examples

The simulator, used in experiments, was developed by the authors and implemented using the C ++ language and the object-oriented programming technique. To develop the simulation model, the process interaction method was used [38]. The developed simulator is capable of determining the blocking probability values for particular traffic classes in a group of cells in 5G systems, in which CAC mechanisms have been implemented. As input data to the simulation program, the capacity of the system is given. Each traffic class is defined by the number of demanded FSUs and the value of the mean service time. Furthermore, a value of *a* is specified, which is numerically equal to the traffic value offered to a single FSU. Based on the values of the above-mentioned parameters, the intensity parameter $\lambda_{s}$ is determined in the simulation program:

(35) $$\sum_{s=1}^{S}\sum_{c=1}^{m}\eta_{s,c}\lambda_{s}/\mu_{c}t_{c}=\sum_{z=1}^{q}k_{z}f_{z}.$$

Depending on the introduced CAC mechanism, the values of reservation limits or thresholds are also entered as input data to the simulator. The exact values of the number of calls generated in each series of simulations are presented in Table 1.

The outlined approaches for assessing the traffic characteristics of a group of cells, collectively managing various traffic streams from multi-service sources, are approximations. To gauge the precision of the proposed solution, we performed a comparison between the computational results and the simulation data. The calculations were executed for a standard system (group) consisting of 7 cells, as illustrated in Figure 4. We posit that a call entering a specified assembly of neighboring cells can be accepted for service if this assembly, represented by any cell within it, possesses an adequate quantity of available resources.

Calculations were carried out for the following structures of a group of cells:

- Group 1:
  − Capacity of particular cells expressed in BBUs: $f_{1}=30$, $f_{2}=35$, $f_{3}=45$, $f_{4}=35$, $f_{5}=45$, $f_{6}=35$, $f_{7}=45$;
  − Traffic classes: m=3, $t_{1}=1$ BBU, $\mu_{1}^{-1}=1$, $t_{2}=4$ BBUs, $\mu_{2}^{-1}=1$, $t_{3}=8$ BBUs, $\mu_{3}^{-1}=1$;
  − Sets of traffic sources: S=2, $C_{1}=\left\{1,2,3\right\}$, $\eta_{1,1}=0.4$, $\eta_{1,2}=0.3$, $\eta_{1,3}=0.3$, $C_{2}=\left\{1,3\right\}$, $\eta_{2,1}=0.5$, $\eta_{2,3}=0.5$;
  − Reservation mechanism: R={1,2}, $Q_{1}=Q_{2}=75\%$ (of total system capacity);
  − Threshold mechanism: T={3}, $p_{3}=1$, $Q_{3,1}=75\%$ (of total system capacity), $t_{3,0}=t_{3}$, $\mu_{3,0}^{-1}=\mu_{3}^{-1}$, $t_{3,1}=6$ BBUs, $\mu_{3,1}^{-1}=1.33$.
- Group 2:
  − Capacity of particular cells expressed in BBUs: $f_{1}=55$, $f_{2}=45$, $f_{3}=55$, $f_{4}=45$, $f_{5}=55$, $f_{6}=45$, $f_{7}=55$.
  − Traffic classes: m=4, $t_{1}=1$ BBU, $\mu_{1}^{-1}=1$, $t_{2}=4$ BBUs, $\mu_{2}^{-1}=1$, $t_{3}=8$ BBUs, $\mu_{3}^{-1}=1$, $t_{4}=11$ BBUs, $\mu_{4}^{-1}=1$;
  − Sets of traffic sources: S=2, $C_{1}=\left\{1,2,3\right\}$, $\eta_{1,1}=0.4$, $\eta_{1,2}=0.3$, $\eta_{1,3}=0.3$, $C_{2}=\left\{1,3,4\right\}$, $\eta_{2,1}=0.5$, $\eta_{2,3}=0.2$, $\eta_{2,4}=0.3$;
  − Reservation mechanism: R={1,2,3}, $Q_{1}=Q_{2}=Q_{3}=75\%$ (of total system capacity);
  − Threshold mechanism: T={3,4}, $p_{3}=1$, $p_{4}=2$, $Q_{3,1}=Q_{4,1}=75\%$, $Q_{4,2}=85\%$ (of total system capacity), $t_{3,0}=t_{3}$, $\mu_{3,0}^{-1}=\mu_{3}^{-1}$, $t_{3,1}=6$ BBUs, $\mu_{3,1}^{-1}=1.33$, $t_{4,0}=t_{4}$, $\mu_{4,0}^{-1}=\mu_{4}^{-1}$, $t_{4,1}=9$ BBUs, $\mu_{4,1}^{-1}=1$, $t_{4,2}=7$ BBUs, $\mu_{4,2}^{-1}=1$.
- Group 3:
  − Capacity of particular cells expressed in BBUs: $f_{1}=80$, $f_{2}=100$, $f_{3}=80$, $f_{4}=100$, $f_{5}=80$, $f_{6}=100$, $f_{7}=80$.
  − Traffic classes: m=3, $t_{1}=1$ BBU, $\mu_{1}^{-1}=1$, $t_{2}=7$ BBUs, $\mu_{2}^{-1}=1$, $t_{3}=14$ BBUs, $\mu_{3}^{-1}=1$;
  − Sets of traffic sources: S=2, $C_{1}=\left\{1,2\right\}$, $\eta_{1,1}=0.6$, $\eta_{1,2}=0.4$, $C_{2}=\left\{2,3\right\}$, $\eta_{2,2}=0.5$, $\eta_{2,3}=0.5$;
  − Reservation mechanism: R={1,2}, $Q_{1}=Q_{2}=75\%$ (of total system capacity);
  − Threshold mechanism: T={3}, $p_{3}=1$, $Q_{3,1}=75\%$ (of total system capacity), $t_{3,0}=t_{3}$, $\mu_{3,0}^{-1}=\mu_{3}^{-1}$, $t_{3,1}=10$ BBUs, $\mu_{3,1}^{-1}=1.4$.

**Figure 5:**
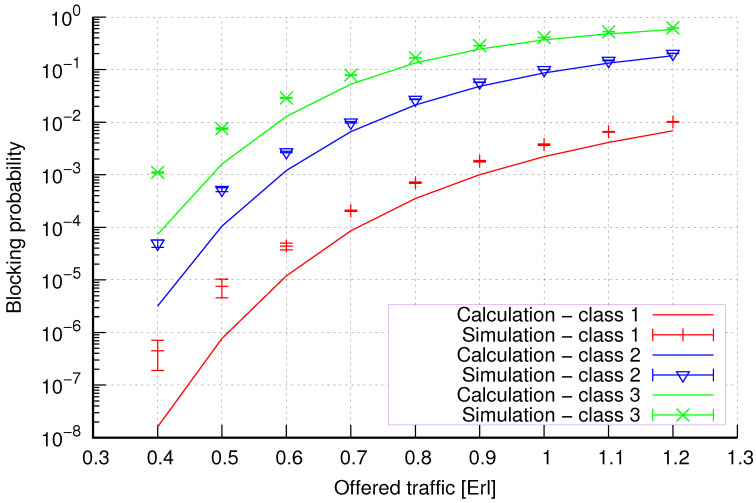
Group 1—method 1; blocking probability in a group of cells with connection handoff.

**Figure 6:**
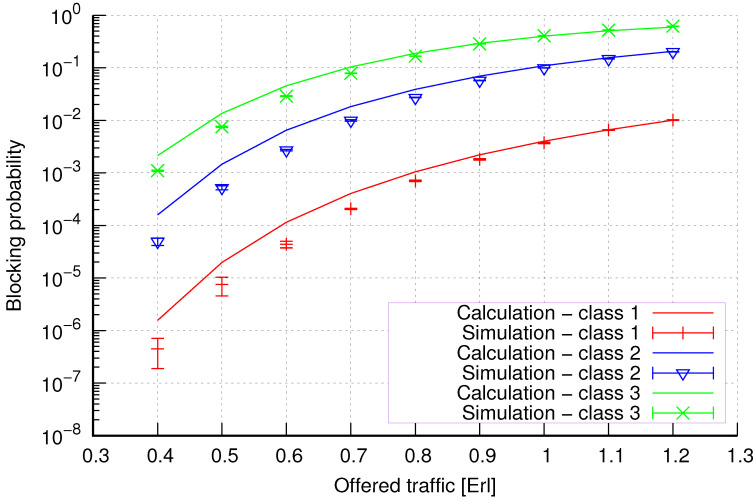
Group 1—method 2; blocking probability in a group of cells with connection handoff.

**Figure 7:**
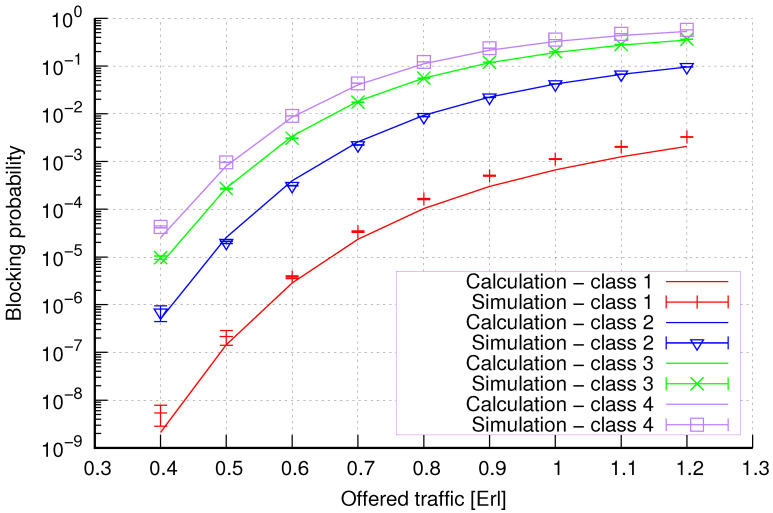
Group 2—method 1; blocking probability in a group of cells with connection handoff.

**Figure 8:**
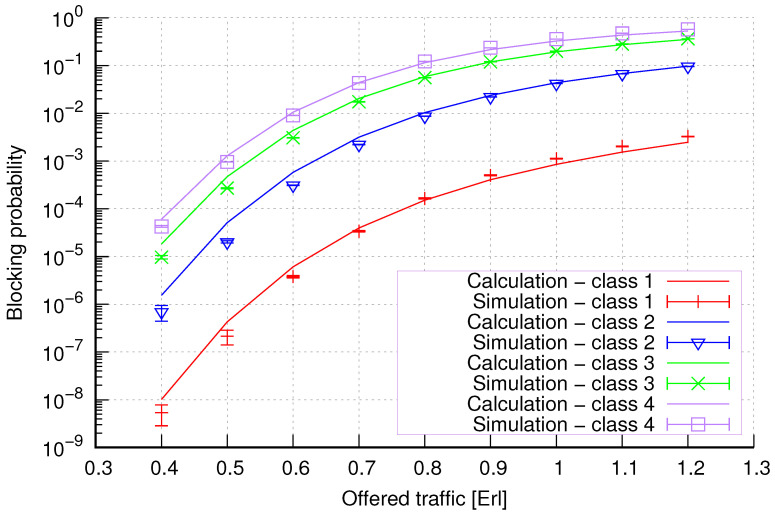
Group 2—method 2; blocking probability in a group of cells with connection handoff.

**Figure 9:**
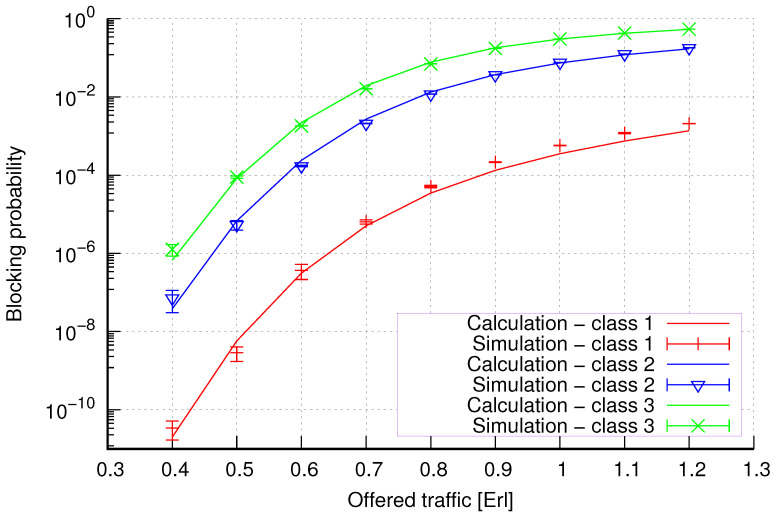
Group 3—method 1; blocking probability in a group of cells with connection handoff.

**Figure 10:**
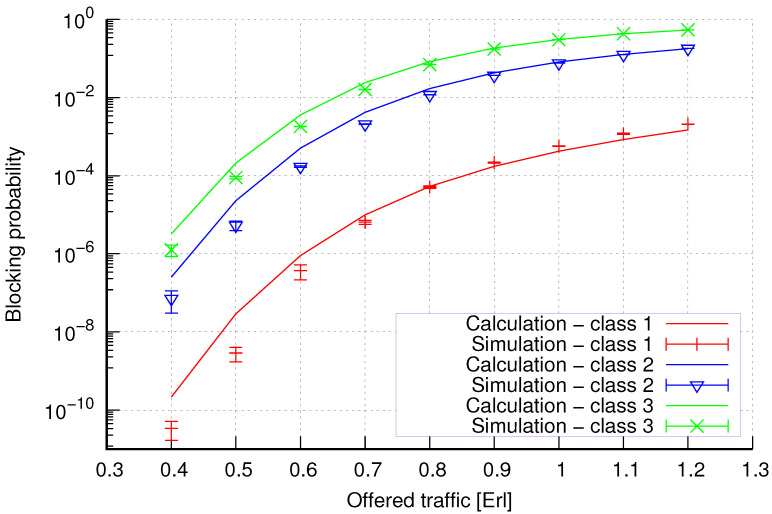
Group 3—method 2; blocking probability in a group of cells with connection handoff.

**Figure 11:**
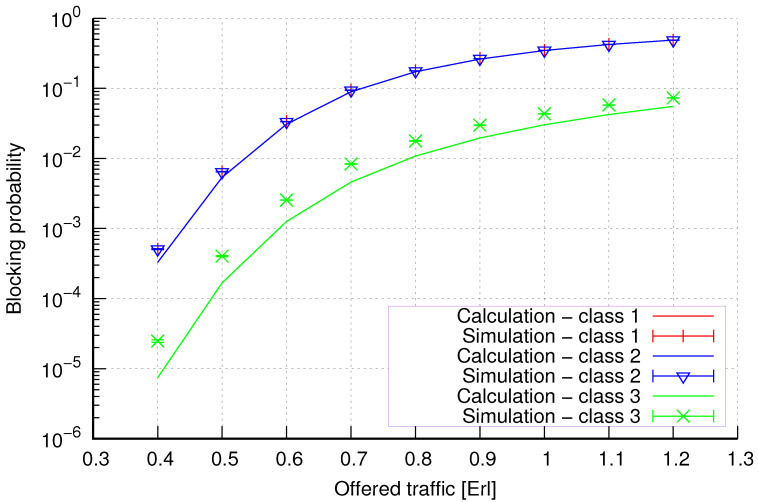
Group 1—method 1; blocking probability in a group of cells with the connection handoff and reservation mechanism.

**Figure 12:**
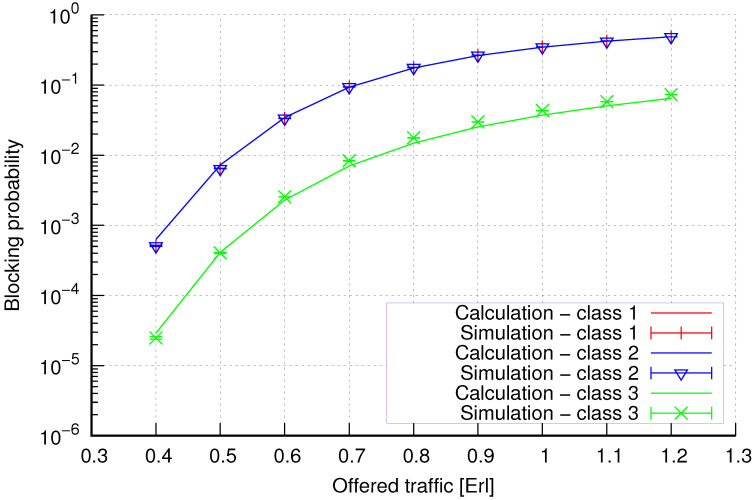
Group 1—method 2; blocking probability in a group of cells with the connection handoff and reservation mechanism.

**Figure 13:**
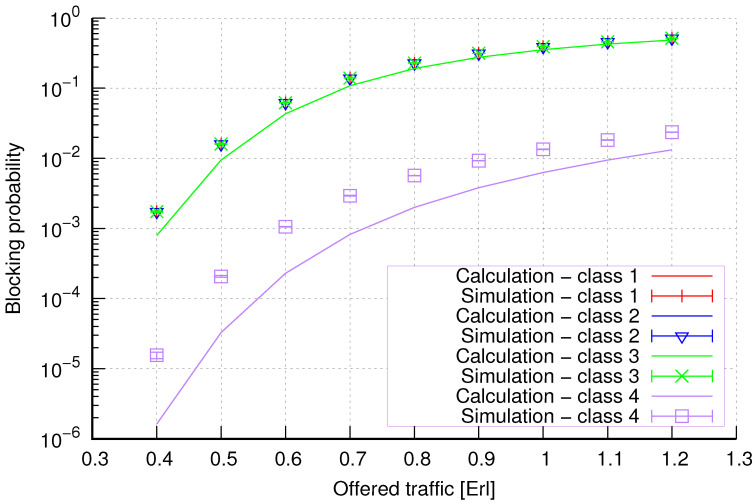
Group 2—method 1; blocking probability in a group of cells with the connection handoff and reservation mechanism.

**Figure 14:**
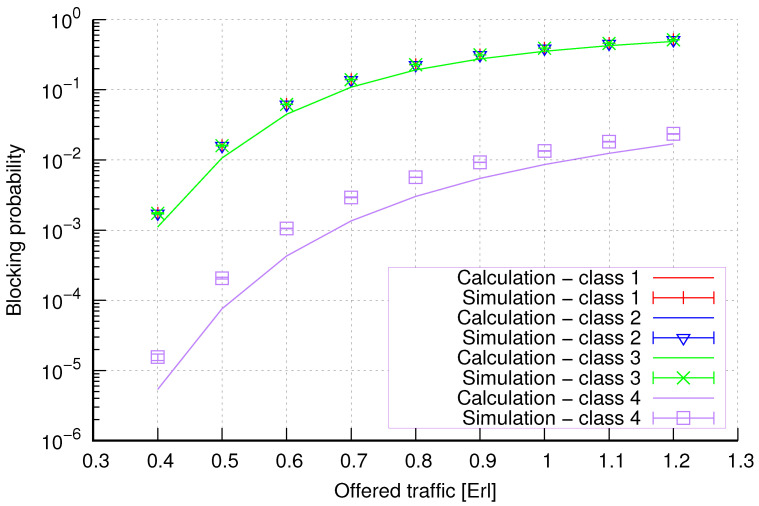
Group 2—method 2; blocking probability in a group of cells with the connection handoff and reservation mechanism.

**Figure 15:**
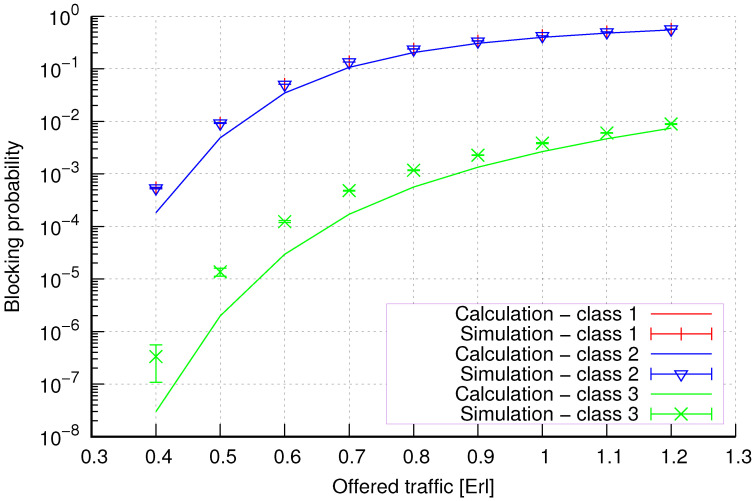
Group 3—method 1; blocking probability in a group of cells with the connection handoff and reservation mechanism.

**Figure 16:**
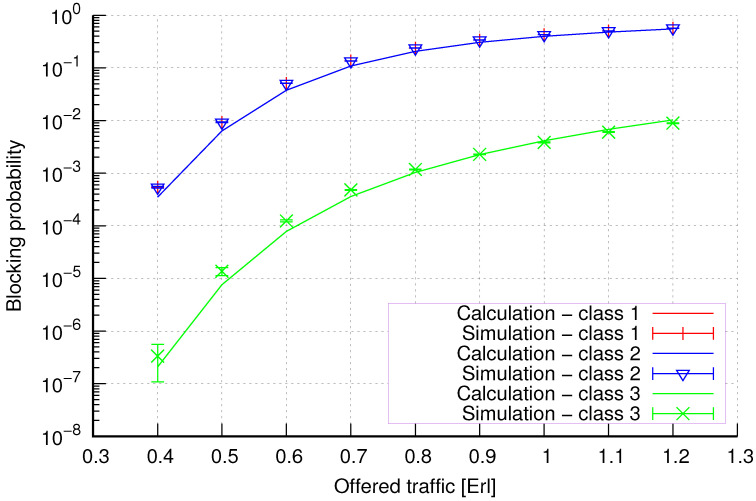
Group 3—method 2; blocking probability in a group of cells with the connection handoff and reservation mechanism.

**Figure 17:**
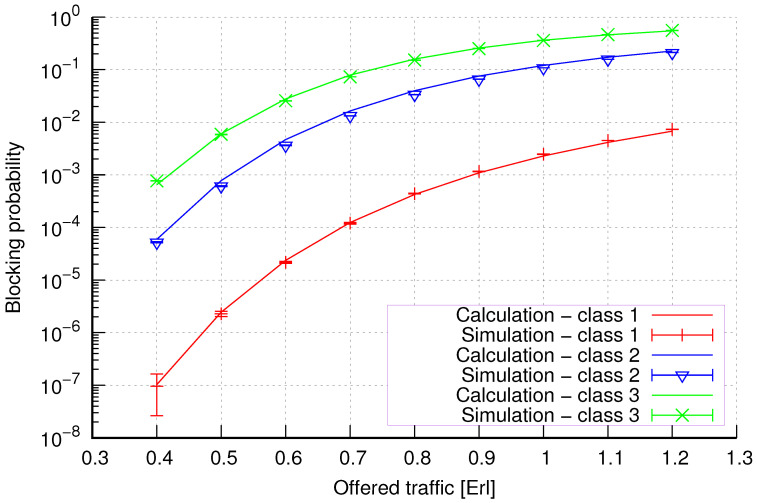
Group 1—method 1; blocking probability in a group of cells with the connection handoff and threshold mechanism.

**Figure 18:**
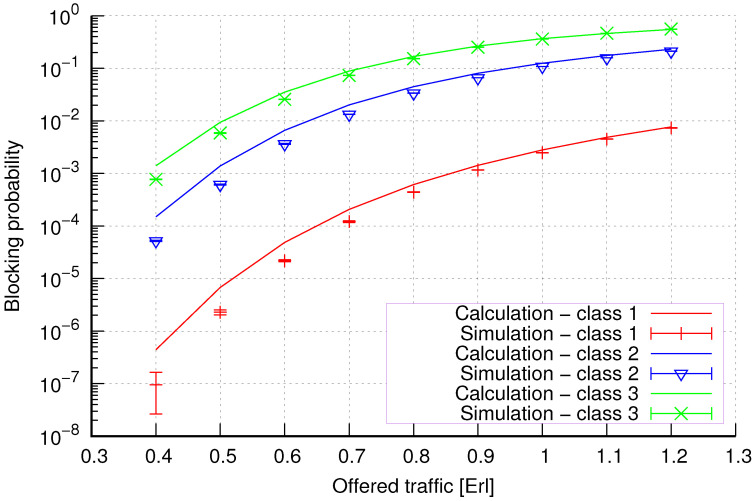
Group 1—method 2; blocking probability in a group of cells with the connection handoff and threshold mechanism.

**Figure 19:**
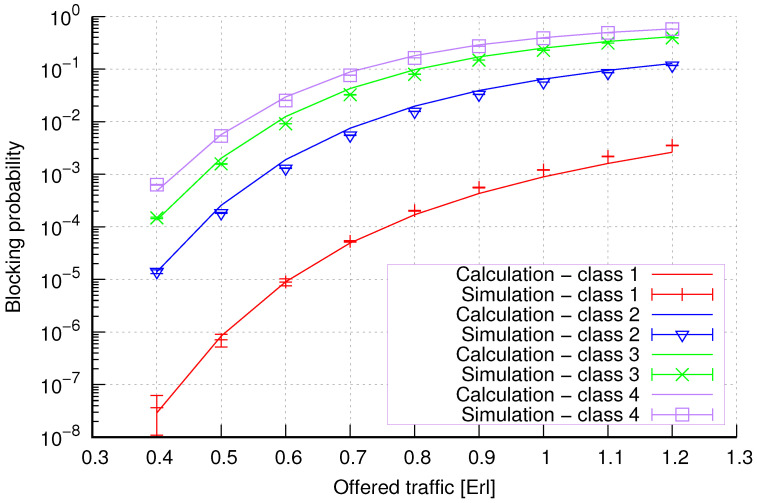
Group 2—method 1; blocking probability in a group of cells with the connection handoff and threshold mechanism.

**Figure 20:**
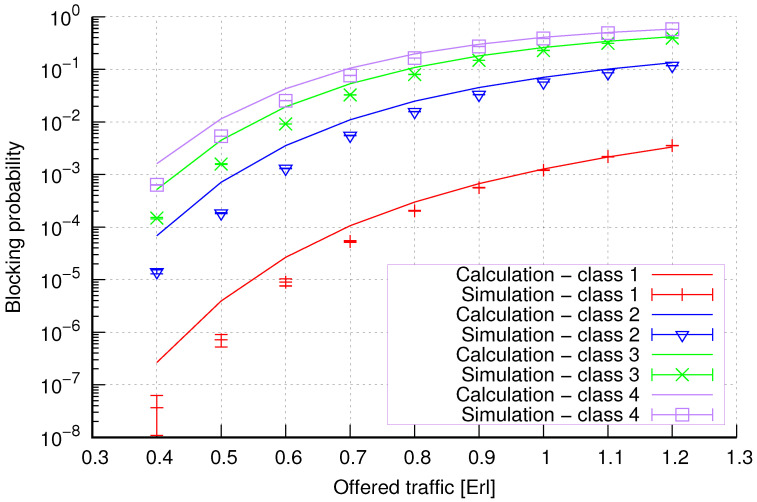
Group 2—method 2; blocking probability in a group of cells with the connection handoff and threshold mechanism.

**Figure 21:**
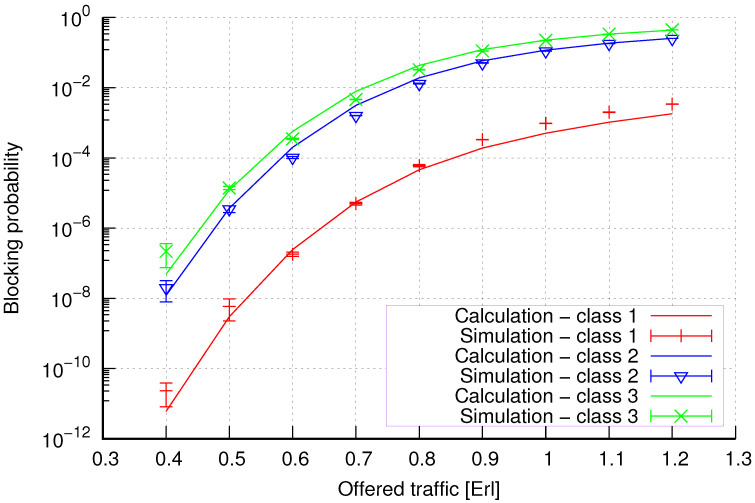
Group 3—method 1; blocking probability in a group of cells with the connection handoff and threshold mechanism.

**Figure 22:**
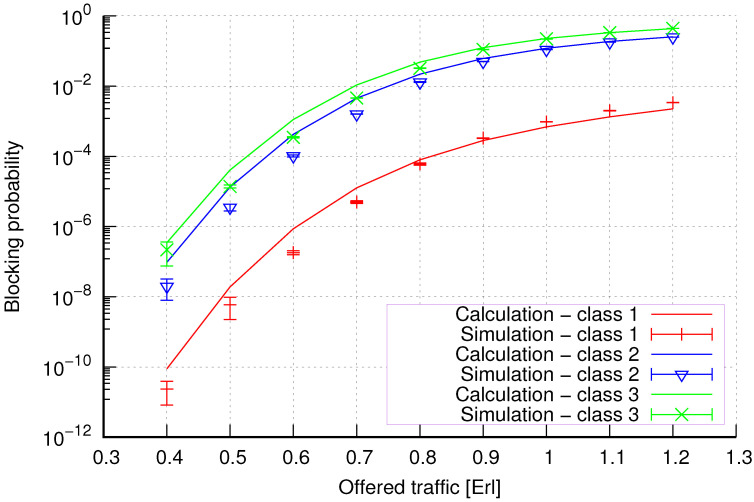
Group 3—method 2; blocking probability in a group of cells with the connection handoff and threshold mechanism.

The results of the calculations and simulations of blocking probabilities for particular classes of call streams in the considered group of seven cells with hard connection handoff mechanisms are presented in Figure 5, Figure 6, Figure 7, Figure 8, Figure 9, Figure 10, Figure 11, Figure 12, Figure 13, Figure 14, Figure 15, Figure 16, Figure 17, Figure 18, Figure 19, Figure 20, Figure 21 and Figure 22. Figure 5, Figure 6, Figure 7, Figure 8, Figure 9 and Figure 10 present the results of blocking probability in systems without CAC mechanisms. In the case of a system with a reservation mechanism, the blocking probability results for individual call classes are presented in Figure 11, Figure 12, Figure 13, Figure 14, Figure 15 and Figure 16. The blocking probability values in systems with threshold mechanisms are presented in Figure 17, Figure 18, Figure 19, Figure 20, Figure 21 and Figure 22. The simulation experiments consisted of carrying out 5 series of simulations of 1,000,000 connections of the least active class for the given system parameters. The simulation results are shown in the figures in the form of appropriately denoted points with a 95% confidence interval. The confidence intervals are calculated using the following formula [39]:

(36) $$\left(\bar{X}-t_{\alpha }\frac{\sigma }{\sqrt{d}};\bar{X}+t_{\alpha }\frac{\sigma }{\sqrt{d}}\right),$$

where $\bar{X}$ is the mean value of *d* results (simulation courses), $t_{\alpha }$ is the value of the *t*-Student distribution for d−1 degrees of freedom. The parameter σ that determines the standard deviation is calculated using the following formula [39]:

(37) $$\sigma^{2}=\frac{1}{d-1}\sum_{s=1}^{d}x_{s}^{2}-\frac{d}{d-1}{\bar{X}}^{2},$$

where $x_{s}$ is the result obtained in the *s*-th simulation run.

Figure 5 presents the blocking probability results in group 1, calculated using method 1. Greater calculation accuracy is achieved for higher system loads, regardless of the traffic class.

Figure 6 presents the blocking probability results in group 1 calculated using method 2. Compared to the results presented in Figure 5, we obtain greater accuracy of the results. However, in this case, better results are obtained for larger system loads.

Figure 7 presents the blocking probability results in group 2 calculated using method 1. The system offers four traffic classes. Therefore, it can be seen that increasing the number of traffic classes does not cause a decrease in the accuracy of the obtained results. In this case, the accuracies for different system loads are at similar levels.

Figure 8 presents the blocking probability results in group 2 calculated using method 2. Compared to the results presented in Figure 7, we achieve similar accuracy. However, in this case, the results are overestimated, which is better from the point of view of system dimensioning.

Figure 9 presents the blocking probability results in group 3 calculated using method 1. Compared to groups 1 and 2, in this case, the capacity of individual cells was increased. We can, therefore, see that this does not reduce the accuracy of the obtained results.

Figure 10 presents the blocking probability results in group 3 calculated using method 2. Compared to the results presented in Figure 9, we obtain lower accuracy results for low system loads.

Figure 11 presents the blocking probability results in group 1 with the resource reservation mechanism calculated using method 1. As we can see, we achieved a reduction in the blocking probability for class 3 at the expense of an increase in the blocking probability for classes 1 and 2. The blocking probability values for class 1 and class 2 calls are equal. In the case of class 3, we obtain results with lower accuracy compared to the results of classes 1 and 2.

Figure 12 presents the blocking probability results in group 1 with the reservation mechanism calculated using method 2. Compared to the results presented in Figure 11, we obtain higher accuracy results for all system loads.

Figure 13 presents the blocking probability results in group 2 with the resource reservation mechanism calculated using method 1. As we can see, we achieve a reduction in the blocking probability for class 4 at the expense of an increase in the blocking probability for classes 1, 2, and 3. The blocking probability values for class 1, class 2, and class 3 calls are equal. In the case of class 4, we obtain results with lower accuracy compared to the results of classes 1, 2, and 3. We can also see that when the number of traffic classes offered increases, the accuracy of the method for class 4 decreases.

Figure 14 presents the blocking probability results in group 2 with the reservation mechanism calculated using method 2. Compared to the results presented in Figure 13, we obtain higher accuracy results for all system loads.

Figure 15 presents the blocking probability results in group 3 with the resource reservation mechanism calculated using method 1. As we can see, we achieve a reduction in the blocking probability for class 3 at the expense of an increase in the blocking probability for classes 1 and 2. The blocking probability values for class 1 and class 2 calls are equal. In the case of class 4, we obtain results with lower accuracy compared to the results of classes 1 and 2. We can also see that when we increase the capacity of particular cells in the group, the accuracy of the method does not decrease.

Figure 16 presents the blocking probability results in group 3 with the reservation mechanism calculated using method 2. Compared to the results presented in Figure 15, we obtain higher accuracy results for all system loads.

Figure 17 presents the blocking probability results in group 1 with the threshold mechanism calculated using method 1. As we can see, in the case of threshold systems for this method, we obtain results characterized by high accuracy, regardless of the system load and traffic class.

Figure 18 presents the blocking probability results in group 3 with the threshold mechanism calculated using method 2. Compared to the results presented in Figure 17, we obtain lower accuracy results for lower system loads.

Figure 19 presents the blocking probability results in group 2 with the threshold mechanism calculated using method 1. Also, in the case of a larger number of traffic classes, the results obtained using method 1 are characterized by high accuracy for all system loads.

Figure 20 presents the blocking probability results in group 2 with the threshold mechanism calculated using method 2. Compared to the results presented in Figure 19, we obtain lower accuracy results for lower system loads and all traffic classes.

Figure 21 presents the blocking probability results in group 3 with the threshold mechanism calculated using method 1. We can also see that for larger capacity cells, method 1 still has high accuracy for all system loads.

Figure 22 presents the blocking probability results in group 3 with the threshold mechanism calculated using method 2. The results obtained confirm that method 2, similar to groups 1 and 2, also exhibits lower accuracy in the case of group 3 for threshold systems when compared to method 1.

So, generally, we obtain greater accuracy for systems without CAC mechanisms using method 2 (Figure 6 and Figure 8). In the case of systems with a reservation mechanism, we also observe greater precision when using method 2 (Figure 12 and Figure 14) for calculations. However, method 1 is also characterized by satisfactory accuracy from the engineering point of view for systems without implemented CAC mechanisms (Figure 5 and Figure 7) and systems with resource reservation mechanisms (Figure 11 and Figure 13). Regarding systems with implemented threshold mechanisms, the results obtained using method 1 (Figure 17 and Figure 19) are more accurate. But, of course, the high accuracy of method 2 also makes it applicable. Therefore, the use of a given method and its accuracy also depend on the type of CAC mechanism implemented in the system.

**Figure 23:**
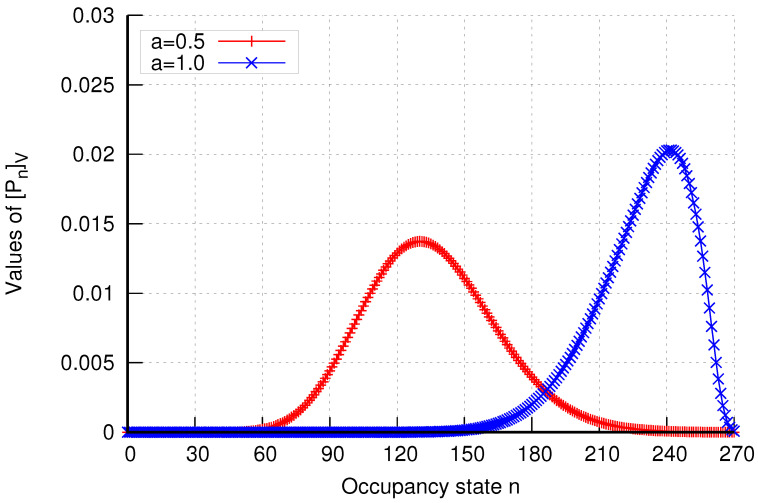
Occupancy distribution in assembly K=1 in group 1 without CAC mechanisms.

**Figure 24:**
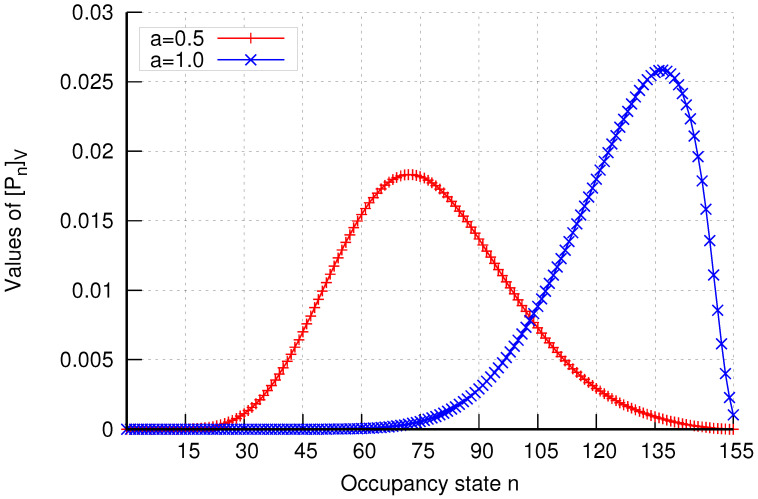
Occupancy distribution in assembly K=6 in group 1 without CAC mechanisms.

Additionally, the analytical model enables the determination of occupancy distributions in assemblies of cells. The results of occupancy distributions in two selected cell assemblies (K=1 and K=6) are presented in Figure 23 and Figure 24. The system’s occupancy distribution allows us to ascertain the most likely load states of the system. Thus, by observing the peak of the graph, we can determine the extent of the system’s load.

In order to evaluate the system’s performance, we can also monitor the number of lost calls for each traffic class in the simulation program. Example results for group 1 are presented in Table 1.

## 6. Conclusions

The paper introduces a system that consists of a cluster of cells that collaboratively handle multiple traffic streams generated by various multi-service traffic sources through connection handoff mechanisms. This study presents analytical approaches to calculate blocking probabilities in the proposed system, considering different CAC mechanisms. These methods leverage an analytical model of a limited-availability group experiencing multi-rate traffic from multi-service sources. A comparison of the results of the analytical calculations with the data obtained from the simulation experiments confirms the high accuracy of the analytical model developed. In most of the obtained results, the relative error does not exceed 10%. The introduced CAC mechanisms make it possible to control the admittance of new calls in the system. In the case of the reservation mechanism, we can influence changes in the blocking probability value. Above a certain reservation threshold, calls of traffic classes that are subject to reservations can no longer be accepted for servicing (their blocking probability increases). The remaining resources are available only to other, privileged traffic classes (their blocking probability decreases). Regarding threshold mechanisms, we notice that changing (reducing) the number of resources allocated to calls of particular traffic classes leads to an increase in the number of handled calls. By reducing the number of resources allocated, we can lower the blocking probability values for individual traffic classes. Therefore, to summarize, thanks to the analytical model of the 5G system with CAC mechanisms, we can assess the impact of the set reservation limit or threshold values on traffic characteristics, including changes in the blocking probability for individual traffic classes. Future research work may concern analytical modeling of 5G systems with other types of mechanisms, ensuring an appropriate quality of service (QoS) level. Attempts will also be made to adapt existing analytical methods to 6G systems. 

## Figures and Tables

**Table 1:** Number of generated and lost calls in group 1 in a particular series of simulations.

Simulation	Generated Calls			Lost Calls		
No.	Class 1	Class 2	Class 3	Class 1	Class 2	Class 3
a=0.4 Erl						
1	8,015,163	2,002,972	1,000,000	0	4	40
2	8,000,646	1,999,606	1,000,000	0	4	36
3	8,003,612	2,001,242	1,000,000	0	6	23
4	7,997,956	1,998,845	1,000,000	1	6	26
5	8,006,855	2,003,639	1,000,000	1	2	42
a=0.5 Erl						
1	8,005,402	2,000,097	1,000,000	3	114	758
2	8,002,535	2,002,714	1,000,000	1	127	807
3	7,990,866	1,998,619	1,000,000	4	112	784
4	8,003,962	2,000,647	1,000,000	4	111	753
5	8,000,179	2,000,384	1,000,000	5	100	662
a=0.6 Erl						
1	8,016,256	2,003,358	1,000,000	84	1414	7098
2	8,001,267	2,003,524	1,000,000	86	1477	7042
3	8,001,351	1,999,157	1,000,000	74	1394	7159
4	8,001,483	1,997,997	1,000,000	73	1516	7055
5	7,997,064	1,997,529	1,000,000	70	1419	7308
a=0.7 Erl						
1	8,005,466	2,000,655	1,000,000	668	9782	35,089
2	7,989,660	2,000,098	1,000,000	735	9672	35,231
3	7,999,643	1,998,351	1,000,000	570	9816	35,163
4	7,998,862	1,996,429	1,000,000	797	9669	35,169
5	8,011,478	2,000,240	1,000,000	643	9626	35,155
a=0.8 Erl						
1	8,016,212	2,003,034	1,000,000	3361	37,033	103,651
2	8,000,809	1,999,740	1,000,000	3304	37,380	104,169
3	8,007,225	2,002,980	1,000,000	3351	36,991	103,743
4	7,989,013	1,998,646	1,000,000	3312	36,748	103,690
5	7,990,865	1,997,921	1,000,000	3348	37,193	104,141
a=0.9 Erl						
1	8,015,925	2,002,674	1,000,000	10,312	91,261	208,430
2	7,996,733	2,000,352	1,000,000	10,123	90,897	209,014
3	8,004,606	1,998,289	1,000,000	10,334	90,849	209,203
4	7,993,835	2,000,331	1,000,000	10,156	90,849	209,182
5	7,993,240	1,997,134	1,000,000	9995	90,761	209,349
a=1.0 Erl						
1	8,006,435	2,000,442	1,000,000	22341	166982	329437
2	8,007,892	2,000,203	1,000,000	22,911	167,469	329,535
3	8,014,370	2,003,343	1,000,000	22,922	168,464	328,232
4	7,999,943	2,001,715	1,000,000	22,415	166,966	329,028
5	7,996,504	1,999,651	1,000,000	22,775	167,821	329,304
a=1.1 Erl						
1	8,018,878	2,003,590	1,000,000	40,424	261,294	443,488
2	7,989,703	1,997,843	1,000,000	41,342	261,824	445,539
3	8,005,807	2,002,445	1,000,000	41,182	260,772	443,122
4	7,996,982	2,000,633	1,000,000	41,035	260,411	444,261
5	8,009,529	2,001,840	1,000,000	40,820	261,568	444,342
a=1.2 Erl						
1	8,019,782	2,003,955	1,000,000	65,725	363,295	545,466
2	8,006,984	2,003,003	1,000,000	65,844	363,668	547,309
3	8,008,821	2,000,908	1,000,000	64,933	362,008	545,762
4	8,003,306	2,001,821	1,000,000	65,496	361,843	545,553
5	8,010,187	2,001,188	1,000,000	65,035	363,342	546,434

## Data Availability

The data presented in this study are available upon request from the corresponding author. The data are not publicly available due to the project’s limitations.

## Abbreviations

The following abbreviations are used in this manuscript:

BBU	basic bandwidth unit
CAC	call admission control
IoT	Internet of Things
LAG	limited-availability group
